# Exploring the Integration of Engineering Design Practices in Tenth-Grade Chemistry Activities

**DOI:** 10.3389/fpsyg.2022.774022

**Published:** 2022-07-13

**Authors:** Abdulwali H. Aldahmash, Yousef F. Alfarraj

**Affiliations:** ^1^Curriculum and Instruction Department, College of Education, The Excellence Research Center of Science and Mathematics Education, King Saud University, Riyadh, Saudi Arabia; ^2^Curriculum and Instruction Department, College of Education, King Saud University, Riyadh, Saudi Arabia

**Keywords:** chemistry textbook, engineering designed practices, new generation science standards, textbooks analysis, science education activities

## Abstract

Chemistry textbooks are the most popular teaching material in schools. They can contribute significantly to the attainment of scientific educational goals. Internationally, educational reforms in science subjects are adopting newer practices such as Engineering Design Processes (EDP) for addressing real-world requirements. This study, conducted in Saudi Arabia, employed a qualitative and quantitative content analysis method to evaluate the level of the EDP incorporated in the tenth grade chemistry textbook and accompanying student’s experiments’ guidebook. The results found the inclusive mean for EDP was 1.05, which indicated incorporation was found to be at level one. This inclusion EDP in chemistry textbooks has been rated as novice or deficient, which indicates that it does not fulfill the majority of the requirements for inclusion as suggested by NGSS.

## Introduction

In the early 19th century, technology and engineering education attracted educators from around the world. For example, the National Academy of Engineering carried out a project called “Engineering in K-12 Education,” that was concerned with the connection between curricular materials related to science, technology, engineering, and mathematics education (STEM) subjects (Pieper and Mentzer, 2013). Since then, engineering education has been integrated with k-12 science curricula in a number of countries (Purzer et al., 2015; Purzer and Quintana-Cifuentes, 2019). For example, the National Science Education Standards (NSES), [National Research Council (NRC), 1996; National Academy of Engineering and National Research Council (NAE and NRC), 2009, p. 4; NGSS, Lead States, 2013; Brand, 2020], adopted engineering design processes and recommended that it be included in science curricula. This integration of engineering design processes in K-12 science education led to reforms in pedagogical, epistemological, and methodological courses and programs for teacher preparation (Benz, 2019; Purzer and Quintana-Cifuentes, 2019). In addition, the NGSS raised engineering design to the same level as scientific inquiry, which was initiated by NSES, by pledging to integrate engineering design into all levels of science education.

To enhance students’ interest in scientific and engineering process, as well as to improve the teaching of science, the NRC further developed its NSES to establish the New Generation Scientific Standard (NGSS). The NGSS were designed to guarantee science education that is grounded in real-world experience (NGSS, Lead States, 2013). Students were expected to be able to investigate and apply important scientific concepts as one of three elements of the NGSS practices. One of the goals of NGSS was also to ensure that students graduated from high school with expertise in engineering design activities and inquiry methods. One way to achieve this would include developing, science textbooks and experiments’ guidebooks with interdisciplinary knowledge, particularly engineering knowledge that would allow students to gain experience rather than memorize concepts and ideas.

The National Research Council (NRC) (2009) highlights the necessity for science training to reflect scientific investigative procedures when generating theories and models and engineering process when building solutions. Ghalia et al. (2016) argues that integrating engineering design processes in K–12 schools can improve student results. They state that incorporating engineering in science education could increase desire to study mathematics and scientific materials. Would also learn to contextualize open-ended issues (Rubel and Mccloskey, 2021). An understanding of the engineering design process could also improve’ knowledge of mathematics and scientific content, and their interest in STEM careers (Berland et al., 2014; Ghalia et al., 2016). The NGSS [National Science Foundation (NSF), 2010; NGSS, Lead States, 2013] was established to reiterate the opinion that scientific courses that “stimulate and create enthusiasm” in STEM would boost the number of students choosing STEM careers. STEM education predominantly emphasizes engineering (Ubuz, 2019). Consequently, there has been an increase in efforts to develop and implement programs like STEM for high schools and K-12 engineering outreach programs (Brand, 2020; Aysun and Ozlen, 2021). However, as Ghalia et al. (2016) point out, although these activities have been successful in introducing the engineering design process to children, instructors remain the most important influence on student outcomes. In addition, Ross et al. (2018) stress, professional development opportunities and knowledge of curriculum-integrated engineering applications are limited, leaving instructors with little assistance to modify their methods.

Educators, in recent years. Have adopted the Engineering Design Process (EDP; Hynes, 2012) as a tool for STEM implementation in a variety of STEM-related topics (Nurtanto et al., 2020). This importance of EDP can be verified by the number of research studies that have been conducted on the efficacy of EDP in STEM education. As a field of study, engineering design can be characterized in many ways. The engineering desirability steps include [National Research Council (NRC), 1996, p. 192; National Research Council (NRC), 2012]: (1) Define the problem, (2) Select a systematic solution, (3) Modeling and Analysis and (4) Repeat the design process. Students’ ability to apply science and mathematics to real-world problems can be considerably enhanced by the implementation of the phases of the EDP (National Research Council (NRC), 1996, p. 192; English and King, 2015; Hafiz and Ayop, 2019). Later in 2012, the National Research Council (NRC) (2012) further developed these phases and recommended eight EDP steps for the K-12 Framework for science education.

Because chemistry textbooks have the potential to influence how students learn as well as how chemistry and science teachers teach and achieve curriculum goals [National Research Council (NRC), 2007, 2012; Niaz and Maza, 2011; Bergqvist and Chang Rundgren, 2017; Kremen et al., 2022]. They influence not only what and how students learn in school, but also what and how science teachers teach science (Niaz and Maza, 2011). Chemistry textbooks play a vital role in schooling. The course structure, as well as the framework for learners’ experience and evaluation, are primarily reliant on textbooks for science and chemistry teachers (Devetak and Vogrinc, 2013; Aldahmash et al., 2016; Nurtanto et al., 2020). Our study is guided by the NGSS and its suggested eight EDP steps [NGSS, Lead States, 2013; National Science Teachers Association (NSTA), 2014, 2018]:

1. Asking questions (for science) and defining problems (for engineering)
2. Developing and using models
3. Planning and carrying out investigations
4. Analyzing and interpreting data
5. Using mathematics and computational thinking
6. Constructing explanations (for science) and designing solutions (for engineering)
7. Engaging in argument from evidence
8. Obtaining, evaluating, and communicating information

We also added another process (redesign) to our analysis rubric, based on the suggestions made by Hirsch et al. (2017). We are convinced that redesign is an important process for students’ mastery of science and chemistry and redesigning activities are essential components of any scientific processes.

### The Rationale of the Study

In 1979, the American University of Beirut’s scientific education experts worked on a reform initiative involving the construction of science curricula in Saudi Arabia. While this reform initiative was completed this year, the enterprise continues to create and execute novel curricular projects. Our qualitative study sought to better understand the impact of the reform initiative in public secondary schools of Saudi. Furthermore, the Saudi science community has grown more concerned that the present curriculum does not adequately reflect the current and future social, cultural, and economic demands of the Saudi society, nor does it adequately satisfy the needs of all students since that time (Alhomairi, 2018). Schools in Saudi Arabia still rely on teacher-centered techniques, which scholars claim, have resulted in a decrease in students’ participation in inquiry-based learning (Al-Ghanem, 1999; Almazroa and Aloraini, 2012). A reform effort to improve the Saudi’s mathematics and science curriculum involved modifications in the McGraw-Hill series textbooks used for primary and secondary education (Al Zughaybi, 2011; Al Zubaidi, 2017). This new curriculum was criticized for being solely inquiry-based, and not incompatible with the standards established by the new NGSS and the science American Association for the Advancement of Science [American Association for the Advancement of Science (AAAS), 1993]. In addition, according to the Trends in International Mathematics and Science Study (TIMSS), this curriculum failed to enhance the achievement of Saudi Arabian students (Mullis et al., 2020; Kremen et al., 2022).

One of the goals of integrating EDP into science courses is to equip students with the knowledge, skills, and abilities to study and apply major scientific ideas in their everyday lives. According to the findings from the Theoretical background, secondary chemistry textbook and experiments’ guidebook that utilized EDP, enabled students to operate like engineers and scientists (Syukri et al., 2018). The efficacy of these books to assist students in practicing EDP skills, on the other hand, has been called into question (Nurtanto et al., 2020). Therefore, one of the goals of our study was to determine the EDP skills contained in science textbooks and the distribution of these skills. We also explored ways to improve the current science curriculum, and identify educational reforms that could significantly improve science and chemistry textbooks. While studies, like ours, is necessary for Saudi Arabian science education, we also believe that our findings will be applicable to education systems in other countries.

The findings of several studies (Tamir, 1991; Dreyfus, 1992; Tamir and Pilar-Garcia, 1992; Green et al., 2006; Hirsch et al., 2017; Lopez and Garlick, 2017) have emphasized the need for adhering to specific rules while examining the content of targeted textbooks. The attention placed on EDP skills to enhance student’s comprehension of science and critical thinking abilities inspired us to delve deeper. As a result, we analyzed chemistry textbooks to understand the integration of EDP in them. One of our concerns was voiced by Dreyfus (1992), who suggested that examining textbooks with such a declared purpose raises a new question: does the textbook match the curriculum’s intentions? This is why we used scientific EDP abilities.

The inclusion of EDP abilities in chemistry textbooks and guidebooks can help students in many ways (Silk et al., 2009; Wendell et al., 2017). First it may help them gain higher test scores in chemistry as well as other science subjects. Another study suggested that EDP would make science more relevant to students as well as increase their scientific reasoning skills. Based on these arguments, it is very important to assess how well school chemistry textbooks include EDP and how they would assist students develop EDP skills.

In our study, we examined the integration of EDP skills in tenth-grade Saudi chemistry textbooks. We selected tenth-grade chemistry textbook because they are taught to students who are in a transition stage, between the middle school and the high school. After graduating the tenth-grade students will have to choose either science or art as their future educational streams. We argue that the inclusion of EDP in these textbooks would increase students’ motivation to select science education.

### Purpose of the Study

The purpose of this study was to examine the tenth-grade chemistry textbook and experiment guidebook for the academic year 2020/2021 in order to identify EDP steps incorporated in their activities. The research questions and sub-questions that guided this study were:

#### The Main Question

To what extent is Engineering Design Process represented in the tenth-grade chemistry textbooks and experiments guidebooks?

##### Sub-questions

- To what extent is the EDP emphasized in the activities provided in both the student’s chemistry textbooks and experiment guidebooks?
- What types of activities could be most effective in promoting EDP among the tenth-grade students?
- Do the EDP skill levels determined in current tenth-grade chemistry textbooks and experiment guidebooks align with the NGSS’s recommendations?

## Research Method

Chemistry textbooks and experiment guidebooks for tenth-grade students were examined using a mixed-method approach that included quantitative data analysis and qualitative content analysis. The units of the analysis were the activities or experiments provided in these books. There were 30 activities and experiments in both the chemistry textbooks and experiments’ guidebook for the tenth-grade students (19 in the textbook and 11 in the guidebook). A methodical approach to examine the targeted textbooks involves examining the activities included in the textbooks Babbie (2001) and White and Marsh (2006) was employed as a guideline in this study. In addition, we looked at the paragraphs, worked examples, figures, and tables to determine whether there were any connections between the practical and theoretical aspects of the document. The processes developed by Babbie (2001) and Krippendorf (2004) were employed to convert raw textual material into standardized codes. This allowed categorization of data for *text-driven analyses, problem-driven analyses, and method-driven analyses* (Nguyen et al., 2020). This was further explained in Part II, titled “Components of Content Analysis,” where the author detailed the many techniques employed in content analyses, starting with their procedural logic and going organically from unitizing to sampling to recording and coding. Thus, in this paper the coding was accomplished by assigning meaning to science concepts in the document and interpreting them in accordance to the analysis cards or rubric. Qualitative data were collected for situations in which EDP abilities were not clearly mentioned in the text or activity but were implied by the phrases used. The coding approach was also used for qualitative data to search for implicit terms and then group them together based on their meaning.

## Materials Analyzed

Two books were analyzed; the first one is the students’ textbook, while the second one was the experiments guide. The activities and experiments, as well as the text and graphs that accompany them, were examined to determine the extent to which EDP was incorporated into the tenth-grade chemistry textbook and experiment guidebook used in Saudi Arabian schools. These books were chosen because they are taught in a grade that functions as a transition year. These books were also chosen because the student in the tenth grade take chemistry for the first time after completing middle school.

The tenth-grade chemistry textbook is 212 pages in length and is divided into five sections and includes 19 activities. The student textbook is divided into the following sections: safety signs, laboratory first aid, a general introduction, how to use the book effectively, general objectives, and a content list. Each chapter of the textbook contains a content list, a launch experiment, and the lessons. Each lesson contains objectives, concepts, new vocabulary, and assessment questions. Numerous lessons incorporate a variety of activities, such as experiments, data analysis laboratories, chemistry laboratories, and problem solving laboratories. Each chapter is concluded with a validated questionnaire.

On the other hand, the tenth-grade chemistry experiment guidebook or experiment guide is 46 pages in length and contains 11 activities named as experiments. It includes an introduction, general objectives, content list, lab safety, instrument, and five chapters. Each chapter contains an experiment, with the exception of chapter two, which contains a three-in-one experiment. Each experiment contains the following components: a conceptual introduction, a problem, objectives, list of tools and instruments required, safety precautions, prior to the experiment, working steps, hypothesis, cleaning and discarding residuals, a table for data and observation, analysis and deduction, error analysis, and real-world chemistry.

The NGSS (NGSS, Lead States, 2013), the National Research Council (NRC) (1996, 2000, 2012), and constructivist theories (Rintaningrum, 2008) all suggest that textbooks and experiments’ guidebooks should be linked, with experiments conducted first and the related content in the textbook discussed and organized based on the results of each activity. According to NGSS students should be treated as scientists when it comes to the design of activities in the students’ science textbook or experiments’ guidebook.

### Instrument

The rubric was developed and the targeted textbook analysis was conducted using the conceptual framework as a guide. Numerous other conceptual frameworks served as the prototype and guide for our study (Chiappetta and Fillman, 2007; Kahveci, 2010; Dunne et al., 2012; Vesterinen et al., 2013; Aldahmash et al., 2016; Hirsch et al., 2017; Aldahmash and Alamri, 2020; Aldahmash and Omar, 2021). Chiappetta and Fillman (2007), for example, used alternative frameworks to outline the inclusion of subject matter content, analyze the difficulty or readability of textbooks or content, and investigate the texts’ epistemological orientation. The current study examined the chemistry textbook and experiment manual for the incorporation of EDP using modified rubrics based on the National Science Education Standards (NSES; National Research Council (NRC), 2012; NGSS, Lead States, 2013). The modifications were carried out using the same techniques as in related investigations (Chiappetta and Fillman, 2007; Kahveci, 2010; Dunne et al., 2012; Mangold and Robinson, 2013; Vesterinen et al., 2013; Aldahmash et al., 2016; Aldahmash and Alamri, 2020; Aldahmash and Omar, 2021). The rubric for this study was designed using the eight NGSS practices (NGSS, Lead States, 2013) and the additional redesign process (Hirsch et al., 2017; see Table 1) included by us (henceforth the term EDP will include all nine practices):

**Table 1:** The rubric for analyzing the inclusion of engineering design process and their levels.

Step	Process	Level 3: (Advanced) or (proficient) meets all the criteria	Level 2: (Developing) meets most of the criteria	Level 1: (Novice) Or does not meet a majority of the criteria	Level 0: (No evidence) Not found
1	Identify the problem	Clearly stated and worded, meets the criteria	Adequately stated and worded, meets most of the criteria	Poorly stated and worded, does not meet a majority of the criteria	Did not include problem statement
2	Framing a design brief	Clearly stated, meets all the criteria and specifications	Adequately stated, meets most of the criteria and specifications	Poorly stated, does not meet the majority of the criteria	Id not include the design brief
3	Research and investigation	The content enables students to do thorough research and investigation of various components of their design	The content just enables students to do adequate research on various components of their design	The content poorly guides students to do research on various components of their design	The content did not include research or investigations
4	Generation of alternative solutions	Generated 3 + thorough sketches of possible design solutions	Generated 2 adequate sketches of possible design solutions	Generated 1 adequate/poor sketch of a possible design solution	Did not include sketches of possible design solutions
5	Choosing the best solution	Thoroughly explained how they objectively chose their solution	Adequately explained how they objectively chose their solution	Did not thoroughly explain solution choice or chose solution objectively	Did not include how they chose the best solution
6	Developmental work	Created thorough sketches, bill of materials, steps needed to create design	Created adequate sketches and bill of materials/steps to create their design	Poor sketch, did not include bill of materials or steps used to create design	Did not include developmental work
7	Modeling (prototyping)	Created a well-designed prototype, allows for testing, Works properly, looks good decent	Created a prototype that can be tested. Works relatively well	Created almost complete prototype, may be able to test, does not work well	Did not include the prototype
8	Testing and evaluating	Thoroughly explained how to test prototype, testing process makes sense stronger	Clearly explained how to test prototype, testing process could be	Did not clearly explain how to test prototype, process not clear	Did not explain how to test the prototype
9	Redesign	Made valid decisions for change/improvement based on test results	Decisions to change were loosely based on test results	Decisions to change were not based on test results	Did not make needed improvements

As shown in Table 1, the rubric contains nine processes, each of which has four alternatives (level 3, meets all the criteria, level 2, meets most of the criteria, level 1, does not meet the majority of the criteria, and level 0, not found). The standards established by the National Research Council (NRC) (2000) and NGSS (NGSS, Lead States, 2013) were used to indicate the level of EDP incorporated in the teaching strategy. The level of openness of a strategy, according to NGSS and NRC can range from level one (teacher-centered) to level four (student-centered), with a preference for levels 3 and 4. According to the National Research Council (NRC) (2000) and NGSS (NGSS, Lead States, 2013) chemistry teaching should incorporate all types of openness levels.

### Reliability of Content Analysis

The authors investigated a sample of the activities in the targeted textbook and experiment guidebook, where two units were analyzed by each of the two raters. Then each rater coded the sampled actions to denote the EDP contained in the studied texts. The kappa formula (Cohen, 1990) was utilized to determine the agreement value. Research (Rubinstein and Brown, 1984) indicates that kappa values between 0.40 and 0.75 are appropriate. The values in Table 2 demonstrate strong inter-rater agreement for all eight EDP abilities contained in the chemistry textbook and experiment guidebook used in this investigation. According to Table 2, the kappa values ranged between 0.877 and (0.559). These values are located in the range between moderate and perfect agreement, which indicates that adequate degree of agreements, has been reached. in this regard, Cohen suggested “the kappa result be interpreted as follows: values ≤ 0 as indicating no agreement and 0.01–0.20 as none to slight, 0.21–0.40 as fair, 0.41–0.60 as moderate, 0.61–0.80 as substantial, and 0.81–1.00 as almost perfect agreement” (McHugh, 2012). These values indicate that the list used in this analysis is adequate for this study and can be used in other similar studies.

**Table 2:** Inter-rater reliability of the analysis of EDP in tenth-grade student’s chemistry textbooks and experiment guidebooks.

Process	% Agreement	kappa	Strength
Identify the problem	93.3	0.877	Almost prefect
Framing a design brief	80.0	0.696	Substantial
Research and investigation	86.7	0.792	Substantial
Generation of alternative solutions	93.3	0.776	Substantial
Choosing the best solution	80.0	0.605	Substantial
Developmental work	80.0	0.679	Substantial
Modeling (prototyping)	80.0	0.559	Substantial
Testing and evaluating	86.7	0.800	Substantial
Redesign	86.7	0.797	Substantial
Total	85.19	0.731	Substantial

#### Levels Assigned Through Calculated Means

To make sense of and draw inferences from the findings, the weighted means and percentages as a measure of central tendency were computed. The interval means were calculated as follows:

First, the range was calculated by subtracting the lowest value from the highest value, so the range is 3–1 = 2. Second, the length of the interval was calculated by dividing the range by the highest value of the scale: (2 ÷ 3 = 0.67).

Finally, the level for each category of the rubric was determined as follows: Not found means were set for the values from 0 to 0.67, Novice means were set from 0.68 to 1.34, Developing means were set from 1.35 to 2.01, and finally, means for the Advanced are set from 2.02 to 3.

## Findings

The results presented in Table 3 indicated that the first practice, “*Identify the problem,”* was the most included EDP. It was directly included in the experiments’ guidebook and indirectly in the activities of the textbook. The practice “*Generating Alternative solutions”* was the least included, all indirectly, with 23.3% of inclusion. The total of the EDP inclusion was found to be 62.58% at the first level, which indicates that 37.42 of the activities did not include EDP. Furthermore, all EDP skills were not properly included in the textbook and the experiment guidebook.

**Table 3:** Frequencies, percentages, means, and standard deviations on the extent of the inclusion of the 9 EDP in the tenth grade chemistry textbook and guidebook.

Process	Advance		Developing		Novice		Not found		Total of included EDP		M	SD	level
	f	%	f	%	f	%	f	%	f	%			
Identify the problem	1	3.3	9	30.0	20	66.7	0	0	30	100	1.37	0.556	2
Framing Design	1	3.3	16	53.3	11	36.7	2	6.7	28	93.3	1.53	0.681	2
Research investigation	3	10.0	10	33.3	11	36.7	6	20.0	24	80.0	1.33	0.922	1
Generating Alternative solution	1	3.3	3	10.0	3	10.0	23	76.7	7	23.3	0.40	0.814	0
Choosing best solution	0	0	5	16.7	5	16.7	20	66.7	10	33.3	0.50	0.777	0
Developmental work	0	0	12	40.0	4	13.3	14	46.7	16	53.3	0.93	0.944	1
Modeling	2	6.7	6	20.0	4	13.3	18	60.0	12	40.0	0.73	1.015	1
Testing and evaluating	3	10.0	12	40.0	6	20.0	9	30.0	21	70.0	1.30	1.022	1
Redesign	4	13.3	11	36.7	6	20.0	9	30.0	21	70.0	1.33	1.061	1
Total	15	5.5	84	31.1	70	25.9	101	37.4	169	62.6	1.05	0.48	1

Regarding the level of inclusion, Table 3 indicates that the inclusion of all EDP skills varied between 1.53 and 0.40, where the practice *Framing Design* recorded the highest level (*f* = 28, M = 1.53) among all other EDP and practice *Generating Alternative solution* recorded the lowest level of inclusion (M = 0.04). It should be noted that all EDP were not included directly or literally in the textbooks. Instead, the inclusion was deduced from the meaning of the sentences or the words. For example, the practice *Identify the problem* was not found in the student’s textbook.

However, all activities included this practice as researchable questions. As an example, in the activity (Experiment: Development of Observation Skills) in the chemistry textbook, Chapter 1, Lesson 3, page 23, the EDP practice *Identify the problem*, was found as a question (*Why are observational skills important in chemistry? Observations are often used to reach conclusions. The conclusion is an interpretation or clarification of the observation*). This was the case in all the activities in the tenth-grade student’s chemistry book. In another example, we used the meaning of the practice *Modeling or Prototype*, provided in the NGSS, to locate this practice in the chemistry textbook. This practice was not found in most of the activities in the targeted chemistry textbook, directly or indirectly.

In some activities, we deduced the inclusion of the practice *Modeling* only from the meaning. For example, in Chapter 4, Lesson 3, the activity (Experiment, observe a reaction that forms precipitate) in page 135, the practice *Create a prototype that can be tested* was included. It worked relatively well. It was written as: *Write a symbolic chemical equation balanced for the reaction between NaOH and MgSO4. Note that most of the sulfate compounds are present as ions in aqueous solutions. The text asks students to: (1) write* *a balanced equation, which requires mathematical modeling.* *(2) Write the complete ionic equation for this reaction, and (3) Determine which ions are spectating, then write the final ionic equation for the reaction*. The general mean of the inclusion of EDP in the activities of the tenth-grade chemistry book was 1.0481. This value indicates that the inclusion was at level one, which indicates that the inclusion did not meet a majority of the criteria. The level one of inclusion is categorized as novice or deficient.

According to Table 4, the total mean of the students’ textbook was 1.0058, while the mean for the inclusion in the guidebook was 1.12. This may show that the inclusion of the EDP in the guide book is higher than in the student’s book. However, the inclusion value for both the textbook and guidebook were at level one.

**Table 4:** Means and standard deviations on the extent of the inclusion of The 9 EDP in both the student’s textbook and experiment guidebook.

Book type		Identify the problem	Framing a design brief	Research and investigation	Generation of alternative solutions	Choosing the best solution	Developmental work	Modeling (prototyping)	Testing and evaluating	Redesign	Total
Textbook	M	1.42	1.37	1.21	0.37	0.53	1.05	0.68	1.16	1.26	1.01
	N	19	19	19	19	19	19	19	19	19	19
	SD	0.607	0.761	1.032	0.684	0.772	0.970	1.057	1.167	1.284	0.54240
Experiments’ guidebook	M	1.27	1.82	1.55	0.45	0.45	0.73	0.82	1.55	1.45	1.12
	N	11	11	11	11	11	11	11	11	11	11
	SD	0.467	0.405	0.688	1.036	0.820	0.905	0.982	0.688	0.522	0.34589
Total	M	1.37	1.53	1.33	0.40	0.50	0.93	0.73	1.30	1.33	1.05
	N	30	30	30	30	30	30	30	30	30	30
	SD	0.556	0.681	0.922	0.814	0.777	0.944	1.015	1.022	1.061	0.47651

In the experiments’ guidebook, the inclusion of some EDP was found to be direct, i.e., the word “problem” was found at the beginning of each experiment. However, the problems were stated as questions and not as statements as recommended by the NGSS. For example, in Experiment 4.1 “*single-replacement reaction*,” in page 33, the problem was stated in the form of two questions; “*The problem What elements replace others in simple substitution reactions? How can the results of these reactions be used to form a chain of chemical activity?*“. In another example, for the practice “*Redesign*,” *in “Experiment 5.2: Mole ratios” page 43,* the book asks students “*what you can do to improve the precision of the measurement of the mole ratio.”* In some cases, the book asked students to redesign the experiment or to design another experiment to achieve the same result.

### Activity Types

As presented in Table 5, there were five types of activities (the activities are labeled similar to the names used in the textbooks or the experiments’ guidebook) identified in the students’ textbook and only one type of activity (labeled experiment) in the experiment guidebook. The mean for all types of activities ranged between 2.00 and 0.00. The activities that represented most of the EDP skills were *experiments*, and the activities that least represented the EDP skills were *problem labs*. However, all the activities had level one EDP skills, which indicates that the inclusion of EDP skills in all activities was at the novice level.

**Table 5:** Means and standard deviations on the extent of the inclusion of the 9 EDP in all types of activities.

Activity type	*N*	Identify the problem	Framing a design brief	Research and investigation	Generation of alternative solutions	Choosing the best solution	Developmental work	Modeling (prototyping)	Testing and evaluating	Redesign	Total
Preliminary experiment:	5	1.80	1.40	1.40	1.00	1.00	1.80	1.00	1.40	2.00	1.42
Experiment	16	1.19	1.62	1.44	0.31	0.38	0.63	0.63	1.38	1.13	0.97
Chemistry analysis lab	6	1.33	1.50	0.83	0.33	0.67	1.17	1.00	1.17	1.33	1.04
Problem lab	3	1.67	1.33	1.67	0.00	0.00	0.67	0.33	1.00	1.33	0.89
Total	30	1.47	1.48	1.33	0.41	0.51	1.04	0.74	1.25	1.42	1.07

The result shows that only the practice (*Identify the problem*) was explicitly included in the experiments’ guidebook, but not in the students’ textbook, even though this inclusion applies only to the title of the Process, not the specification. All other skills were implicitly included in either the students’ textbook or the experiments’ guidebooks. These findings suggest that there is a need to redesign the chemistry textbooks and guidebooks’ activities, in line with the NGSS standards and indicators (NGSS, Lead States, 2013). The activities should also be cognitively related to the rest of the text or the concepts, so that all the content support the development of EDP abilities in students.

## Discussion

The result revealed that the inclusion of all nine EDP abilities in the tenth-grade chemistry textbook and experiment guidebook was categorized as level one. The inclusion of the EDP abilities in the experiments’ guidebook was higher than that in the students’ book. Nonetheless, both books can be categorized as novice or deficient in the inclusion of EDP, which indicates that they did not meet a majority of the NGSS criteria. This result was expected since the chemistry textbook and experiment guidebook used in this study were translated McGraw-Hill series, which were prepared using the scientific inquiry approach, as suggested by NRC standards [National Research Council (NRC), 1996, 2000, 2012], rather than the EDP suggested by the NGSS (NGSS, Lead States, 2013). The EDP is supposed to prepare students to cope with real life experiences after their graduation from general education (Aldahmash and Alamri, 2020; Aldahmash and Omar, 2021). The NGSS recommends using the textbooks to engage students in some form of scientific and engineering design process, such as those included in the analysis rubric. It should be noted that inquiry skills are also not well-represented in the current science (Aldahmash et al., 2016). Analysis approaches for different science curricula have been made and revealed similar results (Ferreira and Morais, 2014; Elmas et al., 2020; Bakken and Andersson-Bakken, 2021; Ferreira and Saraiva, 2021; Vojíř and Rusek, 2022), mathematics (Aldahmash and Alamri, 2020) or chemistry textbooks (Aldahmash and Omar, 2021). Moreover, the inclusion of all inquiry skills was directed toward teacher-centered learning rather than student-centered learning, contrary to the NRC standards [National Research Council (NRC), 1996, 2000, 2012].

Similarly, the results for the second sub-question (what types of activities could be most effective in promoting EDP among tenth-grade student?) asked in this study, revealed that none of the activities were effective in promoting EDP among the tenth-grade students. This was because all activities and experiments exhibited EDP at level one and all activities were novice in the inclusion of EDP skills. This implies that all activities in both the textbook and the experiments’ guidebook should be redesigned to provide students with opportunities to participate in real and authentic science activities. EDP enriched activities would allow students to learn science meaningfully and then acquire all the required skills. To further understand the connection between the activities and the texts the contents of the textbooks were also analyzed. The results indicated that there were some connections between the experiments in the experiments’ guidebook and the text in the student textbook. Unfortunately, there was no indication that the experiments were explorative or EDP enablers. This means that the experiments just serve as factors for confirming the concepts. This situation has to change for students to function as scientists and engineers who are able to cope with real life requirements after graduation. This necessity can be fulfilled by assuring that experiments and other activities used in science textbooks serve as EDP agents. This will also help students build their authentic science knowledge and their EDP abilities (Nurtanto et al., 2020).

The result for the third sub-question (do the EDP skill levels determined in current tenth-grade chemistry textbooks and experiment guidebooks align with the NGSS’s recommendations for engineering design), indicated that the inclusion of all eight EDP skills or abilities were not consistent with the NGSS that recommend constructing all activities to improve engineering designs abilities in students. The textbook and guidebook used in this study lacked EDP practices that could help students build skills required to be scientists or engineers (Syukri et al., 2018; Margot and Kettler, 2019; Nurtanto et al., 2020).

The result also revealed that only the *Identify the problem* ability or talent was directly included in the experiment guide, but not in the students’ textbook, despite the fact that this inclusion only pertains to the skill’s title and not the specification. The textbook for the students and the guidebook for the experiments did not expressly mention any of the other abilities. This indicates that the activities in chemistry textbooks and guidebooks must be modified to meet the NGSS performance expectations [National Science Teachers Association (NSTA), 2016, 2018]. The activities should also be cognitively tied to the text or topics presented in the textbook, so that the entire book content can help students develop EDP skills. Therefore, redesigning the curriculum according to the K-12 science education standards provided by the NGSS is a necessary step to employ these engaging pedagogies. The EDP can be used by science educators to incorporate new projects and technologies into the classroom.

## Conclusion and Implementations

The findings revealed that the EDP skills were included at level one in a tenth-grade chemistry textbook. Level one of inclusion has been labeled as novice or deficient, indicating that the chemistry curriculum fails to meet the NGSS standards and indicators (NGSS, Lead States, 2013). Several barriers prevent effective implementation of EDP in science education. Furthermore, the EDP is a new learning strategy, and many science educators are uninformed about its full potential. These issues, suggest a need for further review studies that explore the nature of the EDP integration in science education. The findings of these investigations can provide useful information that can be applied to improve learning and teaching materials used in STEM education. The EDP’s strength is in the multiple benefits it provides to scientific education stakeholders, but the knowledge required to successfully implement it is still inadequate.

The overall results of the analysis confirmed that the inclusion of the EDP skills in chemistry textbooks does not fulfill current national and international science education standards, including NGSS. Thus, the study may lead to a better understanding of the complete and progressive character of the science curricula. It may pave the way for the improvement of the current science and chemistry curricula. Science instructors can use the EDP practices identified here to introduce new projects and technologies in their classroom. The study’s findings imply that all activities in chemistry textbooks and the accompanying guidebook should be rewritten to encourage authentic science activities that address real-world concerns. Incorporating EDP abilities in chemistry textbooks and guides would also benefit students in a variety of ways. To begin, it may aid students in achieving greater test results in chemistry and other science disciplines (Wendell et al., 2017). Another study suggested that EDP would increase students’ interest in science while also improving their scientific reasoning abilities (Silk et al., 2009). The legitimacy of this process for curriculum creation stems from its long history of use in the EDP. For now, a curriculum that acknowledges the conceptual framework of EDP and is designed to the standards established by the NGSS is required to understand the best practices that can be employed to create engaging pedagogies in science education. Science instructors can use the EDP to introduce new projects and technologies into the classroom. The well-known design method related to such framework is also taught to engineering students as industrial design. As a result, additional study is required to determine the effects of incorporating the EDP into high school chemistry curricula to develop engineering design thinking and conceptual knowledge in high school students. Another future study that focuses on merging the EDP into STEM-based learning in order to strengthen the engineering design skills of chemistry and science instructors could be extremely beneficial.

### Limitation of the Study

This analysis on targeted tenth grade chemistry textbook for the academic year 2021/2022 for The Engineering Designed Processes. However, for the future we would analyzed physics textbooks and activities or experiments guide books for the whole secondary stage.

## Data Availability Statement

The original contributions presented in the study are included in the article/supplementary material, further inquiries can be directed to the corresponding author.

## Author Contributions

AA developed the theoretical formalism, performed the content analytic procedure, statistical analysis, and the numerical simulations, contributed to the final version of the manuscript, and supervised the project. YA proposed the idea, contributed to the development of the theoretical formalism and the final version of the manuscript, and supervised the project. All authors contributed to the article and approved the submitted version.

## Funding

This research was supported by the KSU Deanship for Scientific Research (Group #RG-1441-482).

## Conflict of Interest

The authors declare that the research was conducted in the absence of any commercial or financial relationships that could be construed as a potential conflict of interest.

## Publisher’s Note

All claims expressed in this article are solely those of the authors and do not necessarily represent those of their affiliated organizations, or those of the publisher, the editors and the reviewers. Any product that may be evaluated in this article, or claim that may be made by its manufacturer, is not guaranteed or endorsed by the publisher.
